# Protocol for a Global Burns Research Priority Setting Partnership to agree the most important unanswered questions in international burns care

**DOI:** 10.1136/bmjopen-2022-065120

**Published:** 2022-09-14

**Authors:** Hollie Richards, Robert Staruch, Anni King, Catrin Pugh, Suzannah Kinsella, Jelena Savović, Amber Young

**Affiliations:** 1 Centre for Surgical Research, Population Health Sciences, Bristol Medical School, University of Bristol, Bristol, UK; 2 National Institute for Health Research Bristol Biomedical Research Centre Surgical Innovation Theme, University Hospitals Bristol and Weston NHS Foundation and the University of Bristol, Bristol, UK; 3 Botnar Research Centre, Department of Engineering Science, University of Oxford, Oxford, UK; 4 James Lind Alliance, National Institute for Health Research, School of Healthcare Enterprise and Innovation, University of Southampton, Alpha House, Enterprise Road, Southampton, UK; 5 Population Health Sciences, Bristol Medical School, University of Bristol, Bristol, UK; 6 National Institute for Health Research Applied Research Collaboration West at University Hospitals Bristol and Weston NHS Foundation Trust, Bristol, UK; 7 Children's Burns Research Centre Bristol, University Hospitals Bristol and Weston NHS Foundation Trust, Bristol, UK

**Keywords:** SURGERY, STATISTICS & RESEARCH METHODS, QUALITATIVE RESEARCH

## Abstract

**Introduction:**

Burns affect 11 million people globally and can result in long-term disability with substantial associated healthcare costs. There is limited research funding to support trials to provide evidence for clinical decision-making. Research prioritisation ensures that research focuses on the topics most important to stakeholders, addressing issues of research waste and evidence gaps. The aim of this project is to agree the global top 10 research priorities important to international patients, carers and clinicians from all income status countries.

**Methods and analysis:**

The Global Burns Research Priority Setting Partnership will use James Lind Alliance methods to establish the top 10 research priorities in global burns care. An initial international online multilingual survey will collect candidate research priorities from stakeholders. To increase equity in participation, the survey will also be available via the social media app WhatsApp. Additionally, interviews will be conducted. Data will be analysed to identify and collate research questions and to verify that the priorities are true clinical uncertainties. This list will then be ranked by stakeholders in order of importance via a second online survey. Finally, a consensus meeting will identify the top 10 research priorities.

**Ethics and dissemination:**

The University of Bristol Medical School Faculty Ethical Committee has approved this project. Research into burn care should be prioritised to ensure that funding is focused where most needed. This should be undertaken internationally, to ensure inclusion of the views of professionals and patients from lower income countries, where the incidence of thermal burns is highest. The involvement of the James Lind Alliance will ensure that the methodology is robust and that the patient voice is heard. The final top 10 priorities will be disseminated to funders, governments and researchers internationally to inform future global burns research.

**Strengths and limitations of this study**

- We will use a standardised and proven methodology. The James Lind Alliance have undertaken research prioritisation exercises in more than 100 healthcare areas.
- We have established a steering group of international stakeholders and a wide network of clinical collaborators representing all income status countries.
- The online surveys will be available in eight languages and can also be completed using WhatsApp.
- Language, literacy levels and access to the internet may be barriers to survey access by low-income country participants. We will trial methods to address this limitation with international collaborators.

## Introduction

Burns injuries affect 11 million people globally and 140 000 people in England every year.^1^ Injuries can result in long-lasting functional and psychosocial disability.^2^ Healthcare costs are substantial and are related to long hospital admissions, multiple surgeries and the need for rehabilitation.^3^ Despite the importance to healthcare expenditure, quality of patient life and outcomes, there is a discrepancy between treatment burden and the volume of high-quality evidence in burn care.^4 5^ There is, thus, no consensus on the best approach to current and new treatments,^4^ and subsequently, a wide disparity in care exists between burn services within the UK and globally.^6 7^ This lack of evidence matters, because single-question randomised controlled trials (RCTs) are costly and there is a scarcity of funding available for multiple trials.^8 9^ Evidence to fill clinical knowledge gaps are also not specifically addressed and potential improvements to patient care are missed, as studies do not focus on research areas that are important to patients and clinicians.^10 11^

Research prioritisation ensures that research focuses on questions that are of the most potential benefit to improving outcomes.^12^ This decreases research waste and ensures the most effective use of scarce research funding.^13^ The process involves identifying and prioritising unmet research needs that are important to all relevant stakeholders.^14^ A standardised methodology for research priority setting is provided by the James Lind Alliance (JLA),^15^ which is a non-profit initiative established in 2004 and supported by the UK National Institute of Health Research. The JLA places patients, caregivers and clinicians as central stakeholders,^14 16^ bringing them together into Priority Setting Partnerships (PSP). These Partnerships aim to identify the top 10 most important unanswered questions and research uncertainties.^16^ The process is comprised of three phases: (1) the formation of a steering group; (2) identifying, verifying, refining and prioritising research uncertainties from stakeholders via systematic reviews, surveys and interviews and (3) final agreement through a consensus meeting to agree the top 10 research priorities.

Priority setting exercises are most commonly undertaken within one country.^16^ However, burn injuries occur disproportionately in low and middle-income countries (LMICs), with 70% of all burns occurring in these areas.^17–19^ Not only is the incidence higher in these regions but also access to specialist burn care is limited by geographical and economic constraints.^19^ To address this, the research priority setting exercise for burn care will have a global remit to ensure that the views of patients and clinicians from LMICs are represented.

Focusing research questions on those issues of highest priority to stakeholders will direct future trials to address current evidence gaps. It will reduce research waste^20^ and will provide important new knowledge for researchers, funders and governments. The aim of this project is to work with the JLA to identify the global top 10 research priorities most important to international patients, carers and clinicians.

## Methods and analysis

### Steering group

The Global Burns Research PSP will be led and managed by an international steering group, which has been setup to support the development of this protocol. Guidance on the development and selection of a steering group for research prioritisation exercises is limited. In this project, the aim was to select a steering group that provided expert international multidisciplinary professional experience (eg, surgeons, intensivists, therapists and nursing staff) and those with lived experience of burn care. This was achieved through purposeful selection of individuals via burn professional organisations and patient/survivors through burn support groups and charities. To reflect the global scope of the project, steering group members have been purposively recruited from all continents and from countries with multiple income statuses.

The role of the steering group includes decision-making regarding the scope and remit of the project, contributing to the methodology and data analysis, establishing an international network of partner organisations and individuals to distribute surveys and monitoring the progress of the project throughout. The steering group will meet virtually on a 6 weekly basis. In addition to formal meetings, the steering group members will have access to the online forum ‘Slack’ (https://slack.com/intl/en-gb/) to review and comment on documents on a more regular basis and to ensure that the views of members not able to attend meetings can still be represented and discussed.

#### Context and scope

The Global Burns Research PSP methodology will be developed in accordance with standardised JLA practice.^16^ The scope of this research prioritisation project has been set by the steering group. The scope will be global to reflect the disproportionate incidence of burns injury in LMICs. We have used The World Bank definition of LMICs: ‘*low-income economies are defined as those with a GNI per capita, calculated using the World Bank Atlas method, of $1045 or less in 2020; lower middle-income economies are those with a GNI per capita between $1046 and $4,095; upper middle-income economies are those with a GNI per capita between $4096 and $12,695; high-income economies are those with a GNI per capita of $12 696 or more’.*^21^

The steering group has agreed that the project would look for candidate research priorities in burn prevention, prehospital care and issues around treatment and recovery for patients who have sustained burns that require outpatient or in-patient hospital care. The care of patients with small area burns, that require little to no treatment (Body Surface Area (BSA) of less than 0.5%), will be excluded. Although there are healthcare infrastructure and health and safety regulation disparities between countries that will influence burn outcomes, the care and prevention of burn injuries were felt to be communal to all nations regardless of economy. Suggestions that focus on localised improvements to healthcare infrastructure and health and safety regulations will be excluded, as these are nation specific. Finally, the majority of global burn injuries are caused by thermal mechanisms, and, therefore, it was decided to focus on this area, excluding care for patients with other mechanisms of burn injury (such as chemical or electrical burns) or skin-loss conditions. The treatment and, thus, research priorities for the latter are different to those for thermal burns and these injuries may require their own prioritisation exercise. This JLA protocol for this PSP is available at the JLA website.^22^

In summary, the scope of this PSP is to identify:

- Any unanswered clinical question in international burn care or prevention for patients of any age or gender, with thermal burn injuries of any cause.

The scope will exclude:

- Factors relating to healthcare infrastructure and the economics of provision of care.
- Clinical questions relating to small area burns (defined as injuries of less than 0.5% BSA) not requiring hospital care.
- Care for non-thermal burns (eg, chemical or electrical burns) and non-burn skin-loss conditions.

### Gathering uncertainties from stakeholders (survey 1)

The Global Burns Research PSP will collect clinical uncertainties (candidate research priorities) from patients, carers and multidisciplinary healthcare professionals via online surveys and interviews.

The aim of the initial online survey is to gather uncertainties from stakeholders and will consist of broad open questions with free-text response options regarding which areas of burns care are most important to respondents. The survey will be created using REDCap software that will be hosted by the University of Bristol. REDCap is a secure online application used to capture data for clinical and health research.^23^ The survey will not collect any identifiable data, but respondents will have the option to supply an email address should they wish to be invited to take part in Survey 2. The non-identifiable data will be stored on a separate secure server to these email addresses, so that no survey responses are identifiable.

The online survey is currently available in eight languages based on the predominant languages spoken worldwide and in the regions that have a high incidence of burn injuries. These languages are English, French, (Latin American) Spanish, Brazilian Portuguese, Arabic, Chinese (Simplified), Hindi and Bengali.^24^ The survey is accessible through the project website (www.burnsresearch.bristol.ac.uk/survey1/). Each language version has a dedicated page providing participant information, including what the survey is for, who should take part in the survey, what they are being asked to do and the confidential and anonymous nature of the data being collected. This information will be presented in text and as a plain language animation available in multiple languages. A professional translation service has undertaken the translations of all written and audio materials. As part of the translation process, all written material was proof-read by an independent translator and all surveys will be piloted by native speakers prior to launch to ensure accuracy of contextual translation.

Equity in participation in the survey will be enabled by addressing barriers in countries where internet access is limited by cost and infrastructure. In LMICs, accessing 1 GB of data can cost in the range of 2%–7% of an individual’s monthly income,^25 26^ meaning completion of the survey via the project website may be cost prohibitive. The social media app, WhatsApp, is free and is extensively used in LMICs.^27^ An alternative secondary means of data collection will be offered to participants from LMICs, whereby a version of the survey that can be completed entirely on WhatsApp will be available on request. Data collected by this means will be subsequently entered into REDCap by the project team.

The first survey will be open for approximately 12 weeks, to allow time for awareness to build across different countries and for responses to be submitted.

#### Survey dissemination

A Global Burns Research PSP website (https://burnsresearch.bristol.ac.uk/) has been set up to explain the rationale and scope of the PSP. The online survey will be accessible via this website, WhatsApp and externally via direct weblinks and/or quick response (QR) codes and is available in languages as detailed above. The steering group members and wider group of partner organisations and collaborators will distribute the surveys based on existing burn injury networks and contacts. Methods for survey distributions will include:

- Social media platforms (eg, Twitter (@burnspriorities), Facebook, WhatsApp).
- Personal emails of the steering group to known contacts.
- Burn patient support group websites, newsletters and emails.
- Burns and plastic surgery organisation websites and member distribution lists.
- Emails to lead clinical authors of burns publications in leading burns journals.
- Posters provided in different languages with WhatsApp contact details and QR codes to link to the survey.

There may also be the potential for clinicians or representatives at treatment centres in LMICs to complete surveys on behalf of patients and carers who otherwise would not have access. This will be determined on a case-by-case basis.

#### Participant interviews and literature searches

In addition to stakeholder surveys, in-depth interviews (n=10–20) will be conducted with survivors of burn injuries and clinicians, to gather additional data relating to potential research priorities. The steering group will provide oversight of recruitment, development of interview topic guides and data interpretation. Participants will be recruited through burn support groups, established contacts, burn networks and professional organisations using purposive sampling to maximise variation in demographics. Interviews will be audio recorded and transcribed verbatim. Data will be analysed according to the principles of thematic analysis.^28^ In brief, research topics proposed by interviewees will be reviewed and broad question themes will be established. Topics and questions will then be assigned under the relevant themes in order to establish potential areas for research prioritisation. From these themes, research priorities, written in plain language, will be drafted and added to the longlist of research uncertainties generated by the survey.

Sources of evidence to demonstrate true research questions (evidence uncertainties) will be searched for in parallel with the surveys and interviews. Evidence certainties will be defined as systematic reviews or meta-analyses (or large high-quality RCT) that can draw conclusions on effectiveness of interventions for burn care. These will be explored through a scoping umbrella review of systematic reviews in modern burn care (defined as the last ten years) using Medline, Embase, CINHAHL and the Cochrane Database. If a systematic review has been conducted and has concluded that evidence is available to support an intervention, the certainty of the evidence will be appraised. A second systematic review will search for more recent RCTs, or if a priority is chosen that has no systematic review associated with it. Individual RCTs will only be used if they are not included in a systematic review (eg, when important RCT is published subsequent to the review). Non-randomised studies will not be included. Systematic reviews and RCTs will be assessed pragmatically for the purposes of determining the evidence gaps; the certainty of the evidence for each comparison–outcome combination will be considered more important than a formal rating of the methodological quality of the review. If included reviews have determined the certainty of evidence for their main outcomes, for example, by applying the Grading of Recommendations, Assessment, Development and Evaluations (GRADE) framework,^29^ we will use these evaluations of the certainty of evidence as provided in the review.

If the information required for a GRADE assessment is not reported, such reviews will be considered to be of a lower quality because the lack of the consideration of the certainty of overall evidence will make a review less informative for the purposes of this project. If there are multiple reviews for the same intervention(s), one that has included the assessment of certainty of evidence would be preferable as this is highly informative for developing an evidence gap map (EGM). However, all reviews on the same topic will be inspected and reasons for any discrepancies between findings of reviews considered and recorded. Decision-making regarding evidence certainties will be completed by one researcher and verified by another, with differences of opinion resolved by consultation with a third researcher. We will consider high and moderate certainty evidence as sufficient to allow clinical decisions in burn care, while evidence of low and very low certainty will be recorded as an evidence gap. A list of evidence certainties in burn care, whereby evidence is sufficient to allow clinical decision-making, will be established. This information will be used to develop an EGM where evidence is lacking. EGMs are resources which provide a visual overview of where there is, and where there is not, evidence of reasonable certainty for effectiveness of an intervention.^30–32^

#### Data analysis for survey 1

All non-English survey data will be translated by a professional translation service (Bristol Transcription and Translation Services, Bristol, UK), which will include proof reading by an independent translator. The initial survey is likely to produce a substantial volume of overlapping questions and research uncertainties.^33^ These ‘raw’ questions will be categorised and refined by the PSP core team (AY, HR, RS), with oversight from the steering group, into clear and conceptually distinct research priorities, worded in lay terminology. Similar or duplicate responses will be combined where appropriate, and questions which are outside the scope of the project will be compiled separately. These will not form part of the further prioritisation process, although they will be available for future use on request.

This process will result in a long-list of in-scope verified summary research priorities that capture the themes and topics respondents have suggested, rather than specific research questions. Each candidate priority will be checked against sources of evidence and evidence certainties to determine which questions remain unanswered,^34^ with reference to the outcome of the scoping umbrella review of systematic reviews as described above. A question will be defined as ‘answered’ if evidence exists to allow clinical decision-making (see above).

The steering group will be involved in this process to ensure that raw data are being appropriately interpreted and that finalised research uncertainties can be traced back to raw data in a transparent way. Questions and uncertainties that are not adequately addressed by existing research will be collated for review and refinement by the steering group to produce a final list of summary priorities to progress to the interim priority setting survey.

### Interim priority setting (survey 2)

A second survey will be distributed to patients and clinicians using the methods previously described. This survey will consist of the deduplicated long list of identified and verified research uncertainties. Those respondents to the first survey who chose to provide their email addresses will be sent a link to the second survey. This interim priority setting survey will be available in multiple languages, as described above. Respondents will be asked to select the 10 priorities which are most important to them.

#### Data analysis for survey 2

The priorities selected by clinicians, and those selected by patients and care givers, will be reviewed separately. Separate scores will be kept, to ensure a fair weighting from the different constituent groups. Drawing from each group’s priority list, the 18 highest ranked research uncertainties will be collated for the final prioritisation meeting. The steering group will oversee this process and will discuss any discrepancies with the ranking of questions until consensus is reached.

### Final priority setting and dissemination

The final priority setting will be a virtual workshop facilitated and chaired by the JLA. The workshop will involve patients, carers and clinicians discussing and then ranking the shortlist to determine the top 10 research uncertainties. The steering group will not automatically be involved, in order to ensure that final decisions are made by patients and clinician stakeholders, unbiased by the project team. If it is agreed that steering group members will be involved, only small numbers will take part to provide context for the research priorities. Measures will be taken to ensure that this process is as inclusive and accessible as possible.

The final top 10 research priorities will not be worded as research questions but will be prioritised areas of burns care, which represent evidence gaps considered to be most important by patients and clinicians. The identified priorities can be incorporated at a later point into discrete research questions, which are applicable in different setting, for example, higher and lower income countries and regions with limited access to specialist burns care treatment.

Additional work at the end of the project will be needed to develop the broad priority topics into specific research questions using the Population, Intervention, Comparator and Outcome format and match these topics with appropriate funding sources.^35^ Translating a top 10 priority area into a potentially fundable research project requires mapping, which aspects of the topic remain unanswered and require research, developing a focused research question and designing a suitable project. The UK National Institute of Health Research is automatically informed about the results of PSPs and encourage applications for funding based on a top 10 priority.^36^ For example: in the ‘Blood Pressure in Pregnancy PSP’, priority 9 was: What is the best way to manage pregnancy hypertension (including optimal antenatal and postnatal antihypertensive medication and optimal timing of delivery). The research question that has been funded by the NIHR is: ‘how well blood pressure medicines used to treat high blood pressure in pregnancy work over a short time frame’.

### Patient and public involvement

Patient and public involvement (PPI) refers to research which is carried out ‘with’ or ‘by’ members of the public, rather than ‘about’ or ‘for’ them.^37^ Collaborating with patients and members of the public ensures research answers the most relevant questions for service users and results in a positive impact on society. This project will be coproduced by patients and patient representatives throughout the research cycle. We will include patients with lived experience of burn injuries, service users, healthcare providers, caregivers and members of relevant charities and organisations.

Patients and their representatives will have key roles in the steering group, ensuring their continued involvement in decision-making regarding protocol development, governance, ethical issues and the overall progression of the project. Patients will be involved equally with clinicians in the establishment and prioritisation of research uncertainties by participating in the surveys, interviews and prioritisation consensus meeting. Any patient and public facing project outputs (such as animations and infographics) will be reviewed by PPI members to ensure that the content is clear and relevant.

Evaluation of PPI contributions is vital to assess, and inform patients, of the significance of their contributions. Effective synthesis of PPI evidence will allow for identification of ‘best practice’ and lead to a better understanding of the impact of PPI. To optimise the quality and transparency of PPI reporting within the project, the Guidance for Reporting Involvement of Patients and the Public reporting guidelines will be used.^38^

## Ethics and dissemination

Ethical approval was obtained from the University of Bristol Faculty of Health Sciences Ethics Committee (Ref 9944).

### Dissemination of the final top 10 priorities for burn care research

The final top 10 research priorities from the Global Burns Research PSP will be translated into multiple languages and disseminated to international funders (governmental and non-governmental), stakeholders and global burn-related organisations identified by the steering group. Dissemination routes will include the international network of partners who distributed the surveys, global collaborators, burns charities and support groups and burn academic and clinical networks. Findings will be presented at international academic healthcare conferences related to burns and trauma and published in peer-reviewed open-access academic journals. Additionally, animated videos, infographics and other accessible online content will be developed and disseminated.^39 40^ Social media including Twitter will be used for dissemination of results with translations enabled by the project collaborators.^41–43^

Burn care lacks evidence to support clinical decision-making.^4 44^ This results in variation of care and suboptimal outcomes in some patients.^6 45^ The findings of this PSP will potentially change burn research undertaken globally. It will allow researchers and research funders to focus research, and the scarce resources required to facilitate that research, on topics that are most important to patients, carers and healthcare professionals, thus decreasing research waste.^46^ In this way, research will be focused on relevant and verified clinical uncertainties and funding will be spent wisely.

## Supplementary Material

Reviewer comments

Author's manuscript
