# Supplementary material for: Protocol for a Global Burns Research Priority Setting Partnership to agree the most important unanswered questions in international burns care
Source: BMJ Open. 2022 Sep 14;12(9):e065120. doi: 10.1136/bmjopen-2022-065120 (PMC9476147; doi:10.1136/bmjopen-2022-065120)
Supplement: Reviewer comments [file bmjopen-2022-065120.reviewer_comments.pdf]

PEER REVIEW HISTORY

BMJ Open publishes all reviews undertaken for accepted manuscripts. Reviewers are asked to complete a checklist review form (**http://bmjopen.bmj.com/site/about/resources/checklist.pdf**) and are provided with free text boxes to elaborate on their assessment. These free text comments are reproduced below.

ARTICLE DETAILS

**TITLE (PROVISIONAL)**	Protocol for a Global Burns Research Priority Setting Partnership to agree the most important unanswered questions in international burns care.
**AUTHORS**	Richards, Hollie; Staruch, Robert; King, Anni; Pugh, Catrin; Kinsella, Suzannah; Savović, Jelena; Young, Amber

VERSION 1 – REVIEW

**REVIEWER**	Saidur Mashreky<br>Centre for Injury Prevention and Research Bangladesh
**REVIEW RETURNED**	04-Jul-2022

**GENERAL COMMENTS**	Good initiative. Well-described protocol.
